# The increasing burden and complexity of multimorbidity

**DOI:** 10.1186/s12889-015-1733-2

**Published:** 2015-04-23

**Authors:** Anna J Koné Pefoyo, Susan E Bronskill, Andrea Gruneir, Andrew Calzavara, Kednapa Thavorn, Yelena Petrosyan, Colleen J Maxwell, YuQing Bai, Walter P Wodchis

**Affiliations:** Cancer Screening, Cancer Care Ontario/Action Cancer Ontario, 505 University Avenue, Room 18-14, Toronto, M5G 1X3 Ontario Canada; Institute of Health Policy, Management and Evaluation, University of Toronto, Toronto, ON Canada; Dalla Lana School of Public Health, University of Toronto, Toronto, ON Canada; Institute for Clinical Evaluative Sciences, Toronto, ON Canada; Women’s College Research Institute, Women’s College Hospital, Toronto, ON Canada; Department of Family Medicine, University of Alberta, Edmonton, AB Canada; Ottawa Hospital Research Institute, The Ottawa Hospital, Ottawa, ON Canada; School of Epidemiology, Public Health and Preventive Medicine, University of Ottawa, Ottawa, ON Canada; Schools of Pharmacy and Public Health and Health Systems, University of Waterloo, Waterloo, ON Canada; Toronto Rehabilitation Institute, Toronto, ON Canada

**Keywords:** Multimorbidity, Chronic conditions, Prevalence, Disease clusters, Age, Administrative health data, Ontario

## Abstract

**Background:**

Multimorbidity, the co-occurrence of two or more chronic conditions, is common among older adults and is known to be associated with high costs and gaps in quality of care. Population-based estimates of multimorbidity are not readily available, which makes future planning a challenge. We aimed to estimate the population-based prevalence and trends of multimorbidity in Ontario, Canada and to examine patterns in the co-occurrence of chronic conditions.

**Methods:**

This retrospective cohort study includes all Ontarians (aged 0 to 105 years) with at least one of 16 common chronic conditions. Descriptive statistics were used to examine and compare the prevalence of multimorbidity by age and number of conditions in 2003 and 2009. The co-occurrence of chronic conditions among individuals with multimorbidity was also explored.

**Results:**

The prevalence of multimorbidity among Ontarians rose from 17.4% in 2003 to 24.3% in 2009, a 40% increase. This increase over time was evident across all age groups. Within individual chronic conditions, multimorbidity rates ranged from 44% to 99%. Remarkably, there were no dominant patterns of co-occurring conditions.

**Conclusion:**

The high prevalence of multimorbidity and numerous combinations of conditions suggests that single, disease-oriented management programs may be less effective or efficient tools for high quality care compared to person-centered approaches.

**Electronic supplementary material:**

The online version of this article (doi:10.1186/s12889-015-1733-2) contains supplementary material, which is available to authorized users.

## Background

Chronic disease management has been identified as a key health system concern among developed countries given the rising prevalence and burden of chronic illness [1-3]. According to the World Health Organization, chronic diseases have reached epidemic proportions and constitute the leading causes of death in the world [3]. In Canada, 74% of individuals aged 65+ reported having one or more of 11 chronic conditions in 2008 [4]. Improvements in survival and an aging population are two key reasons that the prevalence of chronic disease and the likelihood of living with more than one condition are expected to continue to rise for the foreseeable future [5,6]. In addition, several lifestyle factors, including tobacco use, physical inactivity, harmful use of alcohol and unhealthy diet have been identified as important contributors to the incidence of chronic diseases and multimorbidity [3,7]. In their study, Fortin et al. found that the likelihood of multimorbidity was associated with the number of unhealthy lifestyle factors [7].

The coexistence of two or more chronic conditions is usually defined as multimorbidity [5,6,8-10]. Empirical studies based on surveys and physician practice records show that multimorbidity is highly prevalent and is the norm, particularly for older adults who are known to be the highest users of the health care system [11,12]. The prevalence can reach between 3% and 98% depending on the setting, data sources and sample characteristics such as age [8,13-20]. Based on registry data collected on patients enlisted in 10 Dutch primary care practices, Uijen and Van de Lisdonk [18] observed that multimorbidity varied by age, sex, and socio-economic class. This study also reported that the prevalence of 3 chronic conditions increased by approximately 60% between 1985 and 2005 while the prevalence of 4 or more conditions increased by 300%. Barnett et al. [21] reported a prevalence of multimorbidity of 23% among patients registered with 314 medical practices in Scotland. While a larger proportion of the population over age 65 experienced multimorbidity, they found that the absolute number of people with multimorbidity was higher among those aged 65 or less [21]. In Canada, Agborsangaya et al. [22] reported that age- and sex-standardized prevalence of multimorbidity was 19.0% among a representative sample of adults 18+ in Alberta. A more recent study in the US also concluded that multimorbidity was common in the population reporting a prevalence of multimorbidity of 23% among their study population in Minnesota [23].

Individuals with multimorbidity have multiple medical and social problems, and both the type and number of conditions exacerbate the consequences of multimorbidity [10,20,24]. Multimorbidity has been associated with lower health related quality of life, higher utilization of health care services and prescribed medications, increased disability, and mortality (2–6, 14, 20–22). However the phenomenon of multimorbidity is not well understood [25-27]. To date most studies have been based on patients enrolled in selected settings [11,28] and population-based estimates are not commonly available.

Although the challenges of efficiently and effectively managing individuals with multimorbidity have been acknowledged, the complexity of the problem is not well understood [5,10,27,29]. In their review of studies evaluating the effectiveness of interventions, Smith et al. [30] concluded that, “interventions targeted either at specific combinations of common conditions or at specific problems for patients with multiple conditions, may be more effective”. The authors also suggest focusing for example on functional decline occurring with multimorbidity, rather than clinical management of specific diseases. As pointed out by Boyd and Fortin [5], some concordant co-occurring conditions may be managed synergistically, whereas discordant conditions increase the complexity of clinical care. In order to better inform approaches to care management, there remains a need to identify the specific clusters of conditions among individuals with multimorbidity, at a population-level.

The aims of this study were to: 1) estimate the population-based prevalence and trends of multimorbidity across all age groups; and, 2) assess the co-occurrence of chronic conditions and describe the most common clusters across multimorbidity groupings. The research also provides an opportunity to assess the usefulness of administrative databases to measure and conduct epidemiological research on multimorbidity.

## Methods

This retrospective study used linked provincial health administrative databases to identify the entire population of Ontario residents aged 0 to 105 years who were registered and eligible for the province’s universal health insurance in 2003 (n = 12,242,273) and 2009 (n = 13,068,845). People were excluded if they fell under the following criteria: had an invalid health card number, were older than 105 years old, died before the index date, or had no contact with the health care system in the last 5 years before the index date (excepting infants). People with no contact with the healthcare system within the past five years are suspected to have either left the province or to have experienced an unreported death.

### Data sources

In Ontario, the costs of all medically necessary care are covered by public health insurance funded from general taxation. This includes all hospital and physician services as well as some home care and long-term care services. Drug coverage is provided to those aged 65 and over and all residents who receive government social assistance payments. All residents of the province are eligible for this medical coverage, and immigrants receive services after a three-month waiting period. Associated provincial health insurance claims databases offer a unique opportunity to identify all individuals who used the health care system and to retrieve information about their medical conditions for inclusion in our cohort. These data are housed and secured at the Institute for Clinical Evaluative Sciences (ICES) under data security and privacy policies and procedures that are approved by the Office of the Information and Privacy Commissioner of Ontario. We used these data to identify persons with chronic conditions who had contact with the healthcare system regardless of where they obtained care. All datasets were linked using unique, encoded identifiers and analyzed at the ICES.

Our cohorts were drawn from a series of linked health administrative databases, not a single insurance file. Ontario’s health care system typically provides services by sector (ie. inpatient, drugs, physician services), and the health administrative databases that capture this service provision are distinct. Therefore, in order to assemble a complete picture of the underlying diagnoses and conditions of Ontarians, various health administrative data were linked using unique encrypted identifiers at the individual level. The Discharge Abstract Database (DAD) consists of data from all hospital discharges in Ontario and the Ontario Health Insurance Plan (OHIP) claims database consists of billing claims for all physician encounters. Additionally, derived chronic condition cohorts developed at ICES using linked data algorithms were also considered [31-37]. These ICES cohorts have been validated and derived from the key databases (DAD, and OHIP claims). The Registered Persons Database (RPDB) was used to identify Ontarians eligible for health insurance coverage and to provide basic demographic information (namely age). Statistics Canada census data were also used to derive population estimates by age and sex in each year [38].

### Identifying the chronic conditions

In order to be considered as cases, individuals were required to have a history of at least one of 16 common chronic medical conditions. We selected conditions based on their clinical relevance and burden, both in terms of cost and outcome (e.g. attributable deaths) as described in previous literature [1-4,24,39,40]. These 16 conditions included: arthritis (excluding rheumatoid arthritis), hypertension, asthma, depression, diabetes, cancer, chronic coronary syndrome (CCS), cardiac arrhythmia, osteoporosis, chronic obstructive pulmonary disease (COPD), congestive heart failure (CHF), renal failure, dementia, rheumatoid arthritis, stroke and acute myocardial infarction (AMI). While this represents a small number of possible conditions experienced by individuals, it captures many of the most substantial conditions from a population-based epidemiological perspective. Six of these conditions (AMI, asthma, CHF, COPD, hypertension, diabetes) were defined based on previously validated population-derived ICES cohorts [31-37]. For the conditions where a derived ICES cohort did not exist, we adopted a similar approach to the derivation algorithms (i.e. at least one diagnosis recorded in acute care, or two diagnoses recorded in physician records within a two-year period) to define the remaining chronic conditions: cancer, cardiac arrhythmia, chronic coronary syndrome, dementia, depression, arthritis (excluding rheumatoid arthritis), osteoporosis, renal failure, rheumatoid arthritis, and stroke. The full set of diagnostic codes used to define the conditions is included as Additional file 1. Multimorbidity was defined as the co-occurrence of two or more of these conditions.

### Analyses

We reported the prevalence of multimorbidity in the Ontario population by age group, level of multimorbidity (2, 3, 4 and 5 or more conditions), and calendar year (2003 and 2009), by looking at concurrent frequencies. Thus, the prevalence was estimated by dividing the number of individuals with multimorbidity by the total population.

We assessed clustering of chronic conditions in two ways. First, for each selected condition, we measured the likelihood of co-occurring with 1, 2, 3 and 4 or more other conditions. The denominator was the total number of individuals with a specific condition and the numerator was the number of individuals with the same condition plus 1, 2, 3 and 4 or more co-occurring conditions. Secondly, we derived the five most common co-occurring clusters of conditions within each level of multimorbidity (i.e. pairs, triads, quartets, and quintets) and measured their prevalence (number of individuals presenting the most common cluster in the targeted level of multimorbidity divided by the number of individuals in this level).

All data analyses were performed with SAS package version 9.3 (SAS Institute, Cary, North Carolina).

The study received ethics approval by the Sunnybrook Health Sciences Centre Review Board. Participants’ consent was not necessary, as we used administrative data.

## Results

Overall, 43% of the Ontario population had a history of at least one of the selected chronic conditions in 2003 and 50.8% had a history of at least one condition in 2009 (Table 1). The proportions varied by age groups, from 24.9% in those younger than 18 years to 92.4% among those aged 90 or more in 2009. The prevalence of multimorbidity was 24.3% in 2009 – an increase of forty percent from 17.4% in 2003 (Table 1). The increasing proportion of people with multiple conditions is a concern for all age groups. For example, 2.2% of people 0 to 17 years and 10.6% of those 18 to 44 years had multimorbidity in 2009 representing an increase of 57.1% and 43.2% respectively compared to 2003 (Table 1). The prevalence of 3 or more conditions also nearly doubled between 2003 and 2009. The increase in multimorbidity is remarkable given that the prevalence of having only one condition remained relatively stable at approximately 26%.

**Table 1:** **Population-based prevalence of multimorbidity in Ontario, by number of common chronic conditions** ^*^ **, age group and year**

**2003**							
**Age groups**	**Ontario Population**	**Prevalence of at least one chronic condition**	**Prevalence of multimorbidity, by degree of multimorbidity**				
			**Prevalence of multimorbidity, 2+ conditions**	**2**	**3**	**4**	**5 + conditions**
0-17	2 794 680	23.2	1.4	1.4	0.1	0.01	0.001
18-44	4 928 716	31.6	7.4	6.0	1.2	0.2	0.05
45-54	1 753 264	51.5	20.4	13.8	4.7	1.4	0.5
55-64	1 213 587	67.6	35.3	20.6	9.3	3.5	1.8
65-74	839 869	82.3	53.6	25.9	15.3	7.3	5.1
75-89	657 855	89.9	68.3	25.7	19.7	11.9	11.0
90+	54 302	93.7	74.6	24.7	20.8	14.3	14.8
All	12 242 273	43.0	17.4	10.0	4.3	1.8	1.3
**2009**							
**Age groups**	**Ontario Population**	**Prevalence of at least one chronic condition**	**Prevalence of multimorbidity, by degree of multimorbidity**				
			**Prevalence of multimorbidity, 2+ conditions**	**2**	**3**	**4**	**5 + conditions**
0-17	2 732 548	24.9	2.2	2.0	0.2	0.01	0.001
18-44	4 951 761	38.6	10.6	8.2	1.9	0.4	0.1
45-54	2 065 338	60.0	27.4	17.2	6.9	2.3	1.0
55-64	1 527 927	76.6	46.6	23.8	13.4	5.9	3.5
65-74	941 352	88.9	66.4	26.1	19.8	11.3	9.2
75-89	775 760	94.0	80.9	21.4	22.0	16.6	20.9
90+	74 159	92.4	83.2	17.1	20.0	18.2	27.8
All	13 068 845	50.8	24.3	12.3	6.2	3.1	2.7

*Arthritis (excluding rheumatoid arthritis), hypertension, asthma, depression, diabetes, cancer, chronic coronary syndrome (CCS), cardiac arrhythmia, osteoporosis, chronic obstructive pulmonary disease (COPD), congestive heart failure (CHF), renal failure, dementia, rheumatoid arthritis, stroke and acute myocardial infarction (AMI).

The prevalence of each chronic condition for each year is shown in Table 2. With the exception of depression and AMI, the prevalence of each condition in 2009 was higher than in 2003. The most prevalent conditions in the cohort as of 2009 were osteoarthritis and other arthritis, hypertension, asthma, depression, diabetes and cancer. However the prevalence of each condition varied with age. Asthma represented the most common condition among children less than 18 years whereas hypertension or arthritis represented the most common conditions among those aged 45+ years (Table 2).

**Table 2:** **Population-based prevalence of 16 common chronic conditions in Ontario, by age group and year**

	**Year**	**Number with condition (prevalence)**	**Prevalence of each condition, by age group**						
			**0-17**	**18-44**	**45-54**	**55-64**	**65-74**	**75-89**	**90+**
**Arthritis (except RA)**	**2003**	1 248 116 (10.2)	1.5	6.8	13.9	18.4	23.7	26.4	21.5
	**2009**	2 728 783 (20.9)	3.1	13.7	27.7	36.5	42.7	48.8	43.7
**Hypertension**	**2003**	1 989 338 (16.2)	0.2	4.6	18.9	35.5	55.4	64.6	57
	**2009**	2 595 152 (19.9)	0.3	5.3	21.6	40.8	60.9	74.3	68.7
**Asthma**	**2003**	1 472 574 (12)	20.6	10.5	8.5	8.6	10.2	10.8	7.8
	**2009**	1 823 345 (14)	20.5	14.2	10.5	10.4	11.2	12.2	9.9
**Depression**	**2003**	1 456 160 (11.9)	2.4	14.2	16.6	14.9	13.9	14.9	12.5
	**2009**	1 409 054 (10.8)	2.2	12.5	14.9	14	11.9	12.3	9.7
**Diabetes**	**2003**	679 519 (5.6)	0.2	2	6.6	12.3	19	18.9	12.4
	**2009**	1 043 016 (8)	0.3	2.6	8.8	16.6	24.7	26.5	17.4
**Cancer**	**2003**	725 971 (5.9)	0.9	3.4	7.1	10	16.4	19.1	12.6
	**2009**	870 906 (6.7)	1	3.5	7.4	11	17.3	21	14.3
**Chronic coronary**	**2003**	375 830 (3.1)	0	0.3	2.1	6	12.8	18	16
**syndrome**	**2009**	625 600 (4.8)	0	0.4	3.1	8.6	17.3	26.7	26.4
**Cardiac arrhythmia**	**2003**	141 599 (1.2)	0	0.3	0.6	1.5	4.1	8.1	8.7
	**2009**	307 447 (2.4)	0.1	0.7	1.5	3	6.7	14.3	17
**Osteoporosis**	**2003**	131 799 (1.1)	0	0.1	1	2.6	4.4	4.8	3.5
	**2009**	287 798 (2.2)	0.1	0.2	1.3	4.8	8.1	10.7	9.6
**COPD**	**2003**	233 844 (1.9)	0	0.1	1.4	3.3	7.8	12.2	11.8
	**2009**	257 012 (2)	0	0.1	1.3	3.3	7.1	11.8	12.4
**CHF**	**2003**	196 169 (1.6)	0	0	0.5	1.9	5.6	13.8	25.1
	**2009**	216 172 (1.7)	0	0	0.5	1.9	5.1	13.2	23.4
**Renal failure**	**2003**	50 680 (0.4)	0	0.1	0.3	0.6	1.5	2.6	2.6
	**2009**	149 234 (1.1)	0.1	0.3	0.7	1.5	3.5	6.9	7.4
**Dementia**	**2003**	88 894 (0.7)	0	0	0.1	0.3	1.4	7.9	22
	**2009**	148 508 (1.1)	0	0.1	0.2	0.5	2	11.2	28.4
**Rheumatoid arthritis**	**2003**	68 279 (0.6)	0.1	0.3	0.7	1.2	1.6	1.8	1
	**2009**	137 729 (1.1)	0.1	0.4	1.2	2.1	2.7	3.3	2.6
**Stroke**	**2003**	56 311 (0.5)	0	0.1	0.2	0.6	1.6	3.6	4.9
	**2009**	124 199 (1)	0	0.1	0.5	1.2	2.9	6.5	9
**AMI**	**2003**	16 848 (0.1)	NA	0	0.1	0.3	0.5	0.8	0.8
	**2009**	13 032 (0.1)	NA	0	0.1	0.2	0.3	0.5	0.7

**Figure 1:**
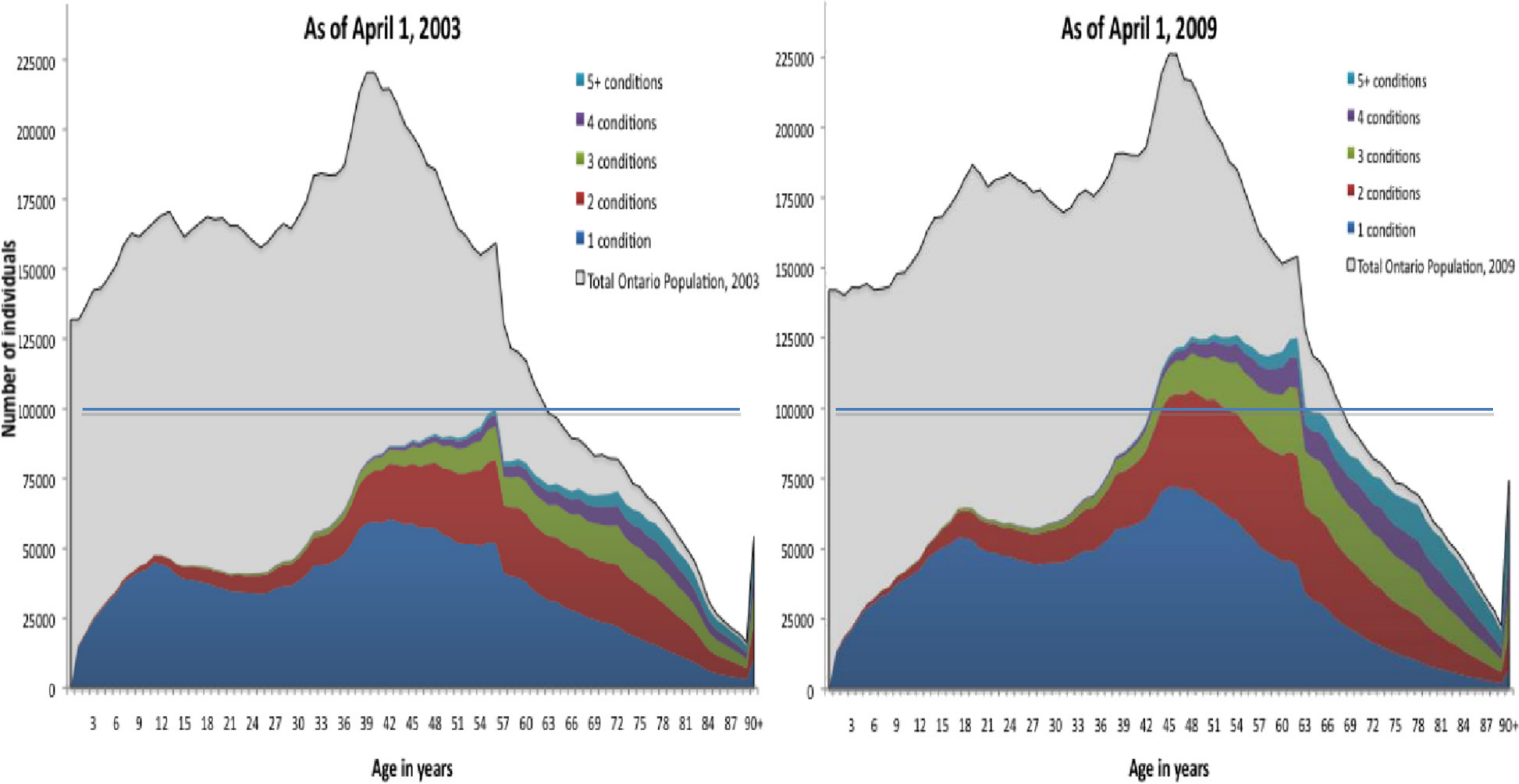
Distribution of the number of individuals with multimorbidity in Ontario across ages, by number of common chronic conditions and year.

Figure 1 compares the trends of multimorbidity across ages between 2003 and 2009. Multimorbidity is highly prevalent in the oldest age groups, where more than 80% of the population had at least two conditions in 2009 (i.e. almost the whole area under the curve for ages 75 or more). While multimorbidity is most frequent in the oldest segments of the population, the prevalence is substantial even among individuals aged 40 or less. Within sub-groups of individuals based on birth year, we observed increasing numbers of individuals with multimorbidity from 2003 to 2009. For example, among individuals who were 56 years old in 2003, 48,101 had two or more conditions; six years later 81,046 of these individuals had two or more conditions. Moreover, in 2003, there was no birth cohort (based on year of birth) that included more than 100,000 people with multimorbidity; but by 2009, there were 20 birth cohorts that included more than 100,000 with multimorbidity (area above the line in Figure 1).

**Figure 2:**
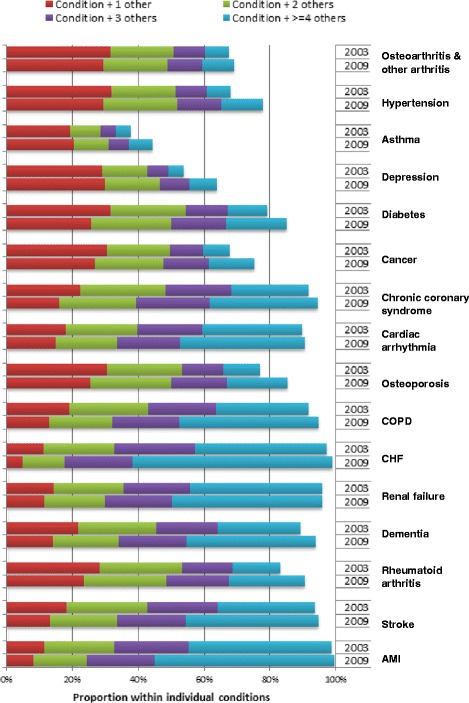
Distribution of the proportion of individuals with multimorbidity in Ontario, within common chronic conditions and by year.

Figure 2 shows the prevalence of multimorbidity within individuals with each chronic condition in 2003 and 2009. With the exception of asthma, which was common in children younger than 18 years and thus most likely to present as a single condition, other conditions were unlikely to occur alone. In 2009, multimorbidity (at least one other condition) within individual chronic conditions ranged from 44% (asthma) to 99% (AMI). In 2009, the proportions of individuals with 4 or more other conditions, ranged from 7.2% (asthma) to 60.8% (CHF).

Table 3 displays the top five most common diseases pairs, triads, quartets, and quintets in 2009. We observed many different combinations of co-occurring conditions within each level of multimorbidity, and as the number of conditions increased, the number of observed clusters increased exponentially. Among those with only two conditions, five possible combinations accounted for 50% of the population with that level of multimorbidity. However, 243 unique combinations of quintets of conditions were required to capture the first fifty percent of individuals with 5 or more conditions. The number of clusters required to include eighty percent of the population increased from 14 (among individuals with two conditions) to 2744 clusters of conditions (among individuals with 5 or more conditions). The five most prevalent clusters accounted for only 6% to 52% of each level of multimorbidity. Even among people with only 2 or 3 conditions, we observed a total of 113 and 443 different clusters represented among the study population (data not shown). Mathematically, with the 16 conditions included in this study, there are 120 and 560 possible combinations of 2 and 3 conditions.

**Table 3:** **Top five frequent clusters of co-occurring chronic conditions among Ontarians in 2009, by level of multimorbidity**

	**Frequency of co-existing chronic conditions, within levels of multimorbidity (Percent of population within level of multimorbidity)**	**Percent of population included in top 5 clusters among those with this level of multimorbidity**	**Number of clusters accounting for:**	
			**50% of those with this level of multimorbidity**	**80% of those with this level of multimorbidity**
**2 conditions (n =** 1 603 837**)**	Hypertension & Arthritis (17.5%);	52.0%	5	14
	Depression & Arthritis (10.0%);			
	Diabetes & Hypertension (9.0%);			
	Asthma & Arthritis (8.8%);			
	Asthma & Depression (6.6%)			
**3 conditions (n =** 816 612)	Diabetes & Hypertension & Arthritis (10.9%);	33.4%	11	45
	Depression & Hypertension & Arthritis (6.8%);			
	Cancer & Hypertension & Arthritis (5.8%);			
	Coronary Syn. & Hypertension & Arthritis (5.0%);			
	Asthma & Hypertension & Arthritis (4.9%)			
**4 conditions (n =** 406 052)	Coronary Syndrome & Diabetes & Hypertension & Arthritis (5.6%);	20.1%	30	127
	Depression & Diabetes & Hypertension & Arthritis (4.0%);			
	Asthma & Diabetes & Hypertension & Arthritis (3.8%);			
	Cancer & Diabetes & Hypertension & Arthritis (3.6%);			
	Asthma & Depression & Hypertension & Arthritis (3.2%)			
**5+ conditions (n =** 348 129)	Asthma & Depression & Diabetes & Hypertension & Arthritis (1.4%);	6.0%	243	2744
	Cancer & Coronary S & Diabetes & Hypertension & Arthritis (1.3%);			
	Coronary S & Depression & Diabetes & Hypertension & Arthritis (1.2%);			
	CHF & Coronary S & Diabetes & Hypertension & Arthritis (1.1%);			
	Asthma & Coronary S & Diabetes & Hypertension & Arthritis (1.0%)			

## Discussion

Our results show that multimorbidity is prevalent in the population of Ontario; it has increased over time and remains a concern in adult populations, including among individuals younger than 65. Among those with at least one of the conditions examined, co-occurrence with multiple conditions is the norm. Multimorbidity manifests differently across individuals, as exhibited by the many different clusters of co-occurring conditions we described. These population-based results for Ontario contribute to epidemiological evidence regarding the prevalence of multimorbidity [4,11,12,21-23] and add unique information regarding population-based trends. These findings have many implications for care management and future research.

### Multimorbidity crosses age groups

Overall, we found that in 2009, one in four Ontarians (24%) had at least two of the 16 conditions selected for the study. This proportion reached more than three in four people (81%) among those aged 75 years or more. The high prevalence of multimorbidity has also been reported in other settings and populations, primarily among older people. Like other studies [12,13,17,18,21,41], we found that multimorbidity significantly increases with age. While there is some (limited) evidence showing that young adults and children may also be affected by multimorbidity [19,21,41], most research has focused on older adults [5,9,18,19,42]. In our study, approximately 1.4% of those aged less than 18 years had multimorbidity, and the most common conditions in this age group were asthma, arthritis (excluding rheumatoid arthritis), depression, and cancer. Among young and middle-aged adults (18–64 years), multimorbidity varied from 7% to 35%, with most common conditions being, arthritis (excluding rheumatoid arthritis), hypertension, asthma, depression, diabetes and cancer. In older adults, besides arthritis, hypertension, and diabetes, chronic coronary syndrome, dementia and congestive heart failure are also highly common and contribute to the elevated prevalence of multimorbidity. While improved care and survival are likely the most important focus among older adults, healthy lifestyles and other behavioural factors are also important areas to focus on particularly among young and middle aged adults [3,7,43]. Physical inactivity, alcohol abuse, and an unhealthy diet have been reported as risk factors for chronic conditions such as cancers, diabetes, hypertension [44]. These factors, which are increasingly prevalent among young people [45,46], might contribute to the observed increases in the prevalence of chronic conditions and multimorbidity. There is a need to continue to advocate for prevention activities and healthy lifestyle and also find better ways to care for individuals with multiple conditions, regardless of their age.

### Complexity of multimorbidity and the need of a patient-centered approach

One of the more interesting findings is that while single-disease prevalence has increased slightly, multimorbidity has increased significantly between 2003 and 2009 in Ontario. The increase in multimorbidity over the 6-year study period was 10 times higher than the increase of any single condition. Very few studies have analyzed trends over time in multimorbidity and/or illustrated trends in single conditions compared to trends in multimorbidity [18,47]. Uijen et al. [18] reported that the prevalence of single chronic disease(s) was stable over the 20-year period of their study, whereas there was a significant increase in multimorbidity, specifically in those with three conditions (67%) and four or more chronic diseases (288%). A similar trend was reported by Paez et al. [47] in their study based on self-reported data; there was a decrease in the prevalence of individuals with single conditions over ten years, compared to an excess of 1.1 and 5.9 percentage points, among those with two and three or more conditions respectively. We found a more remarkable increase in our study, as the prevalence almost doubled over the six-year period, for those with three or more conditions in Ontario. There are many potential reasons for this 40% increase in the prevalence of multimorbidity including unhealthy behaviors as well as improved understanding of conditions and advances in medical technology leading to longer survival and greater aptitude to live with many conditions [3,7,43]. Additionally, the enhancements in the management of administrative databases and improved tracking of diagnoses may continuously contribute to a greater number of individuals identified with any conditions overtime.

Our study also examined the clustering of conditions and found that there was no common typology among individuals with multimorbidity. Because most clinical programs or guidelines for chronic disease management focus on specific and single conditions, there is a growing concern that these programs may not be sufficient or effective for individuals with multimorbidity [5,10,48]. In order to inform the management of individuals with multimorbidity, it is crucial to identify the combinations of conditions among those with multimorbidity, as addressed in this study. Our findings highlighted the challenges in designing effective disease-oriented management programs, as individuals with multimorbidity do not exhibit dominant combinations of conditions. Developing disease management programs for individuals with multimorbidity will be challenging due to the unmanageable number of clusters of medical conditions. Among individuals with four conditions, the largest cluster of conditions represents only 5% of that population, while no distinct cluster has prevalence higher than 1.5% among individuals with 5 or more conditions. This is a daunting prospect for clinicians aiming to manage these individuals in clinical practice. The number and variety of observed clusters suggests that patient-centred care may be a more appropriate generalized clinical approach [10].

### Strengths and limitations

This retrospective population-based cohort study has many strengths and some limitations in regard to the data sources, case definitions and analytical methods, including number of conditions selected or population coverage. The primary limitation could be the use of administrative data instead of definitive clinical information, but this also constitutes a strength for our population-based study. Our study relied on diagnoses recorded in hospitalization data and physicians’ claims to identify people with each of 16 selected conditions. Direct review of medical records has been suggested as the best source for measuring multimorbidity, since they are more comprehensive [11,13]. This is appropriate for individual clinical practices, but population-based analyses require a less resource-intensive approach. Administrative data have been demonstrated to be a relevant alternative [49-51] and a combination of multiple sources of data (hospital and physician) can contribute to produce more reliable estimates. In our study, multiple databases were used to ascertain the cases, including hospital stay (DAD), physician visits (OHIP), and validated disease cohorts.

With appropriate cases definitions, health administrative data can provide valid proxies of clinical status and allow for a suitable estimation of the prevalence or surveillance of chronic conditions [49,51]. An appropriate estimation of the prevalence of multimorbidity depends on the case definition (including selection of conditions), the source of information and the sampling or recruitment strategies [52]. The present study included 16 diagnostic groupings. For six conditions, cases were identified from ICES cohorts based on validated definitions. Validation studies of algorithms used to create ICES registries found sensitivity values from 72% (for hypertension) to 91% (for diabetes). Specificity values varied between 76.5% (for asthma) and 98% (for CHF) [31-37]. A similar approach to case definition was used for non-validated conditions.

In sum, the health administrative databases used in this study have been shown to adequately identify diseases with appropriate algorithms. Moreover the rules for tracking the medical information and the rigorous coding system ensure the accuracy and completeness of the databases. In fact standardized diseases classification and specific fee codes related to the provincial schedule of benefits are being used.

Health administrative databases also offer the advantage of providing information on individuals with multimorbidity at the population level and over time. This study included all of the Ontario population, including children and adults at all ages and found that multimorbidity was a significant concern for younger as well as older populations. The cohort enabled by the use of administrative data also provided sufficient numbers to rigorously assess the clustering of conditions. Thus, we were able to conclude that multimorbidity is a complex matter with few dominant patterns, even among those with two or three conditions. The availability of data in an ongoing manner also creates the possibility of assessing trends and evaluating the longitudinal transitions among individuals with multimorbidity. Future research with these data might detect particular trajectories of disease incidences and lead to identification of risk factors common to particular patterns of multimorbidity. The meaningful findings (increasing trends and age patterns), the multiplicity of data sources, the use of validated registries and the substantial coverage of the population provide some evidence that health administrative databases and the selection of 16 prevalent conditions are useful for measuring and monitoring multimorbidity in Ontario population.

The selection of a limited number of conditions could also be seen as a limitation. However, including all possible conditions is controversial and may be inaccurate since some conditions can be strongly related or constitute risk factors for others [15], leading to an overestimated count. These 16 conditions have a very high degree of overlap with those used in other studies on multimorbidity [24,53]. Most authors usually refer to a limited list of conditions of interest [8,25,29] while others consider an open list of all possible conditions [15,16,18,19,24]. Moreover, when using an open list, only a few and specific conditions contribute the most to the prevalence of multimorbidity [13,14,24,25]. van den Akker and colleagues [19] reported that only a small number of key conditions among a complete list of 335 different diagnoses contributed to the prevalence of multimorbidity, such that adding more conditions to the key conditions did not significantly alter the overall count of individuals with multimorbidity. Fortin and colleagues [11] concluded that it is preferable to use a list of at least 12 chronic diseases for measuring multimorbidity. Our selection of conditions was based on population impact, which is affected by population prevalence and the impact of the conditions on health. The population health impact of these conditions is well supported by previous studies [1-4,24,39,40]. In their review of multimorbidity indices, Diederichs et al. [54] recommend the inclusion of at least 11 of the most common diagnoses. All of these conditions were included in our study. Other researchers could decide to include additional conditions but that is unlikely to affect the general conclusions offered by the present study. In sum, the selection of the 16 conditions for our study constitutes a strength, considering the objective of studying the prevalence of multimorbidity at the population level. The inclusion of more conditions would likely not change the findings on the relative increase in multimorbidity over time or the lack of common clusters. However, extending the number of conditions (e.g. including more mental and physical impairments resulting from multiple chronic conditions) would likely lead to slightly higher estimates of the prevalence of multimorbidity and a greater complexity (ie. level of multimorbidity and clusters). As shown by McLean et al. [41] there is a higher likelihood that considering more mental health conditions would impact the prevalence and complexity of multimorbidity in younger ages.

Our work and that of others emphasizes the need for a more comprehensive approach to understanding multimorbidity, which needs to take into account a greater number of psychiatric conditions and social factors which influence coping and management [54].

An appropriate understanding of multimorbidity also needs to address the factors associated with this phenomenon. Though our study focused on the differences related to age, there are many other factors, including sex and socioeconomic status that contribute to the burden of multimorbidity [18,21,22,41,55]. In future analyses, the impact of sex and socioeconomic status on the prevalence and complexity of multimorbidity will be explored in greater detail.

## Conclusion

We found that multimorbidity was highly prevalent in Ontario and increased significantly between the two time periods examined. As expected, age was strongly associated with multimorbidity. Perhaps unexpectedly, we found great diversity rather than dominant combinations of chronic conditions, suggesting that single disease-oriented management programs may be less effective tools for high quality care compared to person-centered approaches. The high and increasing prevalence of multimorbidity observed in this study confirm the need for health care providers to focus on this issue and highlights the importance of evaluating the impact of multimorbidity on health outcomes, costs and quality of care. Finally, there is a growing need to address care management, patient experience and costs. It is important to explore the common problems (e.g. pain management, functional decline) associated with multimorbidity, in order to inform decision making for recommended practice approaches. Constituting one of the most significant challenges for health care in the 21^st^ century, more research is needed and is taking place to evaluate the determinants, outcomes and costs of multimorbidity.

## Additional file

Additional file 1:
**List of diagnosis codes for defining the 16 selected conditions.**
