# Supplementary material for: The increasing burden and complexity of multimorbidity
Source: BMC Public Health. 2015 Apr 23;15:415. doi: 10.1186/s12889-015-1733-2 (PMC4415224; doi:10.1186/s12889-015-1733-2)
Supplement: Additional file 1: — List of diagnosis codes for defining the 16 selected conditions. [file 12889_2015_1733_MOESM1_ESM.docx]

**Appendix A: List of diagnosis codes for defining the 16 selected conditions**

| **Condition** | **ICD 9 / OHIP** | **ICD 10** |
| --- | --- | --- |
| AMI | 410 | I21, I22 |
| Arthritis - Osteoarthritis | 715 | M15-M19 |
| Arthritis - Other Arthritis (Synovitis, Fibrositis, Connective tissue disorders, Ankylosing spondylitis, Gout Traumatic arthritis, pyogenic arthritis, Joint derangement, Dupuytren’s contracture, Other MSK disorders) | 727, 729, 710, 720, 274, 716, 711, 718, 728, 739 | M00-M03, M07, M10, M11-M14, M20-M25, M30-M36, M65-M79 |
| Arthritis - Rheumatoid arthritis | 714 | M05-M06 |
| Asthma | 493 | J45 |
| Cancer | 140-239 | C00-C26, C30-C44, C45-C97, |
| Cardiac Arrythmia | 427.3 (DAD) / 427 (OHIP) | I48.0, I48.1 |
| CHF | 428 | I500, I501, I509 |
| COPD | 491, 492, 496 | J41, J43, J44 |
| Dementia | 290, 331, 797 (OHIP) / 290.0, 290.1, 290.3, 290.4, 290.8, 290.9, 294.1, 294.8, 294.9, 331.0, 331.1, 331.2, 797 (DAD) | F000, F001, F002, F009, F010, F011, F012, F013, F018, F019, F020, F021, F022, F023, F024, F028, F03, F051, F065, F066, F068, F069, F09, G300, G301, G308, G309, G310, G311, R54 |
| Depression | 311, 300, 296 | F32, F33, F412, F480 |
| Diabetes | 250 | E08 - E13 |
| Hypertension | 401, 402, 403, 404, 405 | I10, I11, I12, I13, I15 |
| Osteoporosis | 733 | M81 M82 |
| Renal failure | 403,404,584,585,586,v451 | N17, N18, N19, T82.4, Z49.2, Z99.2 |
| Stroke | 430, 431, 432, 434, 436 | I60-I64 |
| Coronary syndrome  (excluding MI) | 411-414 | I20, I22-I25 |
